# Caudal optogenetic spinal stimulation promotes better therapeutic outcomes than rostral optogenetic stimulation following a cervical spinal cord injury

**DOI:** 10.3389/fnins.2026.1939104

**Published:** 2026-10-20

**Authors:** R. J. Myers, V. Dang, M. N. McGiffin, M. L. Trinh, S. E. Mondello

**Affiliations:** 1 Department of Rehabilitation Medicine, University of Washington, Seattle, WA, United States; 2 Center for Neurotechnology, Seattle, WA, United States

**Keywords:** axonal growth, cervical hemicontusion, forelimb recovery, optogenetic stimulation, rehabilitation

## Abstract

**Purpose:**

Spinal stimulation, including optogenetic stimulation, is a promising therapeutic approach for promoting functional recovery after spinal cord injury (SCI). To date, most studies have delivered stimulation caudal to the level of the lesion. Therefore, there is a lack of understanding of how spinal stimulation *rostral to* the lesion may affect recovery. Here, we investigate the effects of optogenetic spinal stimulation rostral to the lesion site on forelimb functional recovery and axonal growth in a rat model of cervical SCI.

**Methods:**

Female Long-Evans rats received a C4 hemicontusion, intraspinal injections of an optogenetic viral vector rostral to the injury site, and a micro-light-emitting diode (μLED) implant for optogenetic stimulation. Beginning 6 weeks post-injury, rats received 6 weeks of optogenetic or sham stimulation alongside forelimb reaching rehabilitation. Forelimb reaching success and growth-associated protein 43 (GAP43) percent area were compared with those in our previous study, which evaluated an identical paradigm, but with optogenetic stimulation positioned caudal to the injury.

**Results:**

The present results indicate that rostral stimulation did not enhance forelimb recovery on the forelimb reaching task and that forelimb reaching success was significantly higher in the caudal optogenetic stimulation group compared to the rostral stimulation group. Additionally, rostral stimulation did not enhance axonal growth within the cervical spinal cord compared with sham stimulation, whereas caudal optogenetic stimulation promoted significantly more axonal growth than rostral optogenetic stimulation.

**Conclusion:**

Overall, these results indicate that caudal optogenetic stimulation promotes a greater therapeutic impact than rostral optogenetic stimulation.

## Introduction

Spinal stimulation is currently one of the more promising treatments under investigation for treating spinal cord injury (SCI), with most studies using electrical stimulation caudal to the lesion site (Inanici et al., 2018; Inanici et al., 2021; Samejima et al., 2022; Moritz et al., 2024; Harkema et al., 2011; Angeli et al., 2014; Gerasimenko et al., 2015; Kasten et al., 2013). In addition, our group and others discovered that optogenetic spinal stimulation caudal to the lesion promotes significant functional recovery in rat models of cervical SCI (Mondello et al., 2023; Alilain et al., 2008).

The majority of spinal stimulation studies position the stimulator *caudal* to the injury site to increase excitation of the downstream premotor and motor neurons (Harkema et al., 2011; Rejc et al., 2017; Rejc et al., 2016; Rath et al., 2018; Jilge et al., 2004; Angeli et al., 2018). However, previous work demonstrates that increased activation of axotomized neurons upregulates both regeneration-associated genes and growth factors and promotes axonal plasticity (Mondello et al., 2014; Al-Majed et al., 2000; Al-Majed et al., 2000; Al-Majed et al., 2004). Consistent with this finding, optogenetic stimulation of the motor cortex rostral to an SCI enhanced axonal outgrowth at the lesion site (Deng et al., 2021) and promoted corticospinal tract regeneration beyond the lesion (Ma et al., 2025). Another study using electrical stimulation found that electrical stimulation of the parent nerve above the site of a nerve transection significantly enhanced the speed and accuracy of nerve reinnervation (Al-Majed et al., 2000). Taken together, these results suggest that stimulation of the spinal cord rostral to the lesion site may be beneficial for building new circuitry and promoting functional recovery.

In the current study, we aimed to evaluate and compare the therapeutic efficacy of optogenetic stimulation rostral to a cervical spinal cord injury with optogenetic stimulation caudal to the injury. To achieve this, we delivered optogenetic stimulation to the spinal cord one segment rostral to a cervical hemicontusion injury using the same paradigm as our prior study of caudal optogenetic stimulation (Mondello et al., 2023). This included evaluating performance on the forelimb reaching task and quantifying upregulation of growth-associated protein 43 (GAP43) throughout the cervical spinal cord. These findings will allow us to identify strategies for optimizing therapeutic spinal optogenetic stimulation after SCI.

## Methods

All animal procedures were approved by the University of Washington Institutional Animal Care and Use Committee (4618-01), and optimal housing conditions were ensured in accordance with established animal welfare guidelines for the duration of the study, including maintaining a temperature of 68–75 °F, a 12-h light/dark cycle, and clean air ventilation. Ten female Long-Evans rats (250–350 g; Inotiv, West Lafayette, IN) were divided into two groups: *rostral optogenetic stimulation (n = 5)* and *rostral unstimulated sham (n = 5)*. We compared the results for these rats with those from our previous study, in which rats received *caudal* optogenetic or sham stimulation (Mondello et al., 2023). Rats received a cervical hemicontusion (C4) and an injection of an optogenetic virus ipsilateral and rostral to the lesion site (C3; Figure 1). At 4 weeks post-injury, rats received a micro-light-emitting diode (μLED) implant over C3 to deliver optogenetic light stimulation. Two weeks later, rats began receiving optogenetic stimulation that continued for a total of 6 weeks. Then, rats were perfused, and spinal cord tissues were processed for markers of injury size and axonal growth. Rats from our previous study underwent an identical experiment, except the viral injections and implant were positioned at C6, caudal to the lesion (Mondello et al., 2023) (Figure 1). Please refer to our prior publications for details on the methods below (Mondello et al., 2023; Mondello et al., 2021).

**Figure 1:**
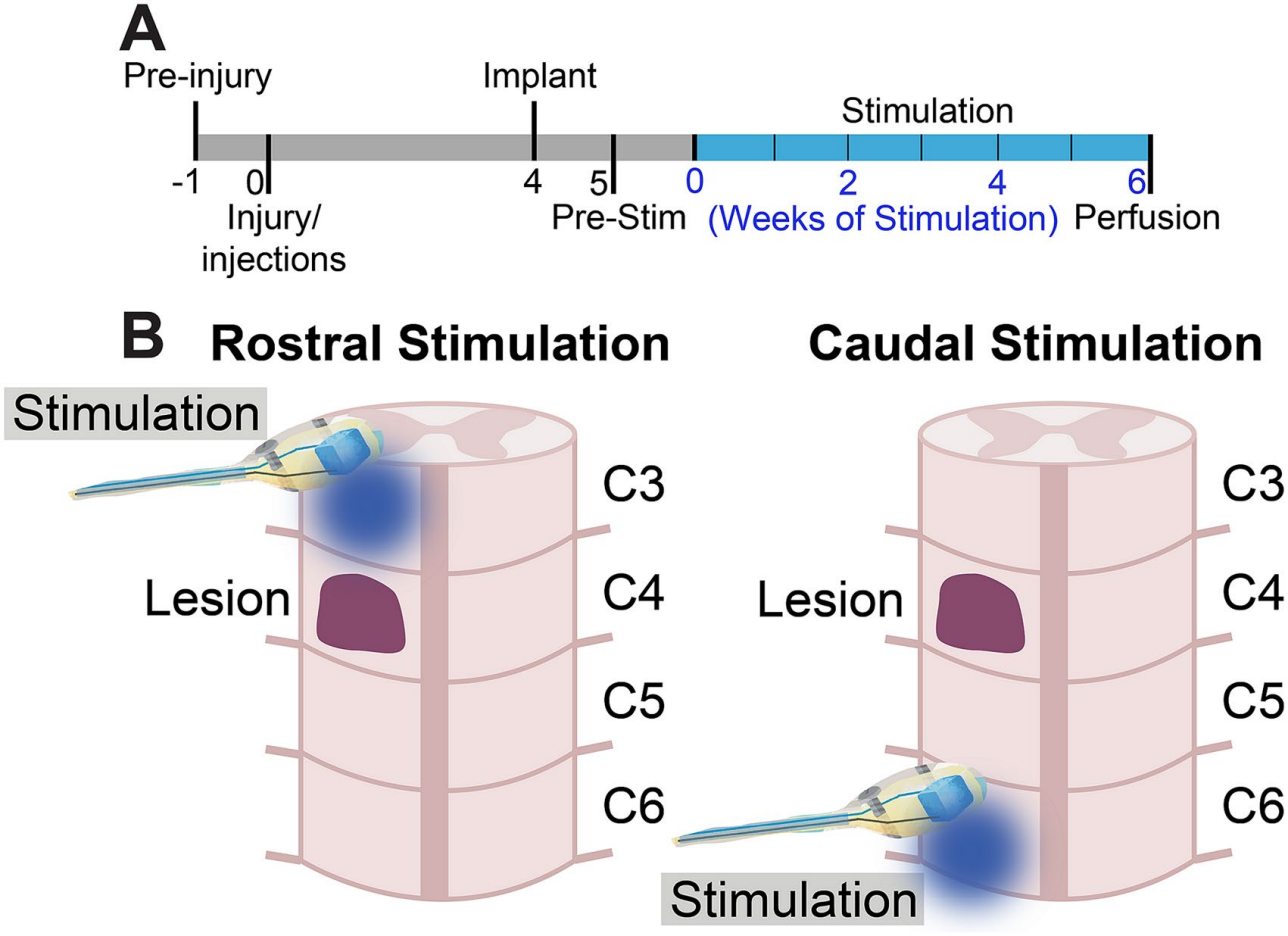
Experimental paradigm. **(A)** The experimental timeline highlights the timing of key steps throughout the study for all groups. **(B)** The location of optogenetic stimulation relative to the lesion in the rostral and caudal stimulation paradigms is depicted.

### Spinal cord injury and intraspinal injections of channelrhodopsin-2 (ChR2) adeno-associated virus (AAV) vector

Anesthetized rats received a C4 hemicontusion injury following a hemilaminectomy over spinal segment C4 using the Infinite Horizon Impactor (150 kdyne; Precision Systems and Instrumentation, LLC, Fairfax Station, VA). During the same procedure, rats received intraspinal injections of the optogenetic vector *AAV2-hSyn-ChR2-YFP* (Deisseroth Laboratory, UNC Vector Core, Chapel Hill, NC) at ipsilateral spinal segment C3. Two 1.5-μL deposits of the vector were injected in all animals. The same viral vector, injection depth, and injection volume were used for the caudal sham and stimulation groups, but at ipsilesional C6 (Mondello et al., 2023). All rats received a subcutaneous injection of slow-release buprenorphine for pain management.

### μLED implant procedure

For the rostrally stimulated or sham groups, anesthetized rats received an incision over C2–T2, and a small amount of vertebral lamina was removed over caudal C3. The μLED implant was positioned over ipsilesional C3 and anchored to the remaining lamina using bone screws (J. I. Morris). Slow-release buprenorphine was administered for analgesia once the surgery was complete. The same steps were completed for the caudal sham or stimulated groups; however, the implant was positioned over ipsilesional C6.

### Daily behavioral training: skilled forelimb reaching task (FRT)

The forelimb reaching task was performed 15 min per day, 5 days per week for the entirety of the study. Weekly videos of the forelimb reaching task were used to determine the percentage of successful reaching attempts. Five weeks post-injury was considered the “pre-stimulation” time point used for comparison during all subsequent periods.

### Therapeutic optogenetic stimulation

Beginning 6 weeks post-injury, stimulation was delivered at a pulse frequency of 4 Hz and a pulse width of 5 ms, with trains of stimulation delivered for 5 s on and 15 s off for 1 h. The light intensity was set at movement threshold within a range of 25–150 mW and was determined prior to each stimulation session (Mondello et al., 2021). During the first week of stimulation, optogenetic stimulation occurred four times per week. For the remainder of the 5 weeks of stimulation, optogenetic stimulation occurred once per week, similar to our previous study (Mondello et al., 2023). Both treated and sham rats were connected to a stimulation cable during their forelimb reaching rehabilitation. Only the treated animals received optogenetic stimulation. A portion of the stimulation period occurred while the rats were performing the forelimb reaching task to guide the formation of new, functionally relevant circuitry. These parameters were the same as in our previous caudal optogenetic stimulation study (Mondello et al., 2023).

### Histology

Rats were transcardially perfused using 0.9% sodium chloride, followed by 10% formalin. Tissue was cryopreserved, blocked, and cross-sectioned at 40 μm (Mondello et al., 2023). To evaluate spared tissue at the lesion epicenter, every 12th section from the lesion site was washed in phosphate-buffered saline (PBS) containing 0.1% Triton X-100 for 10 min (0.1% PBS-T; Sigma-Aldrich, St. Louis, MO). The sections were then washed in PBS for 5 min and incubated in a Nissl solution for 20 min (1,350; Molecular Probes, Eugene, OR). After another 10-min wash in 0.1% PBS-T and two 5-min PBS washes, the tissue was counterstained in a myelin dye solution for 20 min at room temperature (1,200; Molecular Probes). Afterward, the tissue was washed in PBS, mounted onto a slide and coverslipped. The section with the greatest amount of damage from the injury was then imaged using a Zeiss Axio Zoom V16 microscope (Zeiss; Jena, Germany). FIJI, an open-source software package (Schindelin et al., 2012), was then used to quantify the amount of spared tissue at the lesion epicenter and determine the area of spared tissue within the ipsilesional hemicord (Samejima et al., 2022).

To evaluate the spread of optogenetic viral transduction for the stimulated rats, every 12th section from the rostral or caudal regions relative to the lesion was mounted onto a slide and imaged. A tracing of the gray matter laminae was overlaid on the section with the greatest amount of viral transduction for each rat, and the laminae containing viral transduction were identified. The proportion of rats from each group with transduction located in specific laminae was compared.

*Immunohistochemistry*: Similar to our previous study, every 12th section was investigated for GAP43 immunoreactivity (1:250; Millipore; AB5312, Burlington, MA) (Mondello et al., 2023). The total area of GAP43-labeled fibers was determined using FIJI (Schindelin et al., 2012). Four sections per spinal area were analyzed. Each section was imaged using a Zeiss Axio Zoom V16 microscope (Zeiss) at 0.52 pixels/μm using the exposure range indicator to normalize exposure across sections. In FIJI, each image was divided into ipsilateral and contralateral hemicords using the region of interest (ROI) feature and converted to 8-bit grayscale. Next, the background was subtracted (40 pixels), edges were found, and the image was made to be binary. After running the Analyze Particle program, the total area of pixels was saved for each ROI, indicating the fiber area. The fiber areas for each region and rat were averaged, and then the average fiber area for each group was determined. In addition to being completed in the rostral stimulation and sham groups, this analysis was also completed in the caudal stimulation and sham groups using tissue from our prior study (Mondello et al., 2023).

### Experimental design and statistical analysis

Non-parametric tests were used to assess statistical significance. Fisher’s exact test was used to compare transduced laminae between rostrally and caudally stimulated rats. The Kruskal–Wallis test was used for mean comparisons in the FRT and GAP43 analyses. Pairwise comparisons using Dunn’s test were completed if the null hypothesis was rejected. All tests were performed using SPSS software version 28.0.0.0 (IBM, Armonk, NY).

### Transparency, rigor, and reproducibility

Experimenters performing all behavioral and image analyses were blinded to the treatment groups following best practices for spinal cord injury research. All animals were randomized to experimental conditions, and rats with infections beneath the implant or signs of significant autophagia were excluded from the study.

## Results

### Location and spread of optogenetic viral transduction were similar across rostrally and caudally stimulated groups

The location of optogenetic viral transduction was qualitatively and quantitatively evaluated within the ipsilateral gray matter at C3 and C6 in the rostrally and caudally stimulated rats, respectively. The laminae containing yellow fluorescent protein (YFP) labeling were identified for each rat. Overall, the rostrally and caudally stimulated groups had similar locations of viral transduction, with most rats having transduction within laminae III–X (Figure 2). Although more rats had transduction in laminae I and II in the caudally stimulated group, these differences were not significant (*p* = 0.175 and 0.175). A similar pattern was observed for laminae V, VIII, IX, and X, with the caudally stimulated group having more rats with transduction in these areas. These differences were also not significant (*p* = 0.455, 0.455, 0.455, and 0.182, respectively).

**Figure 2:**
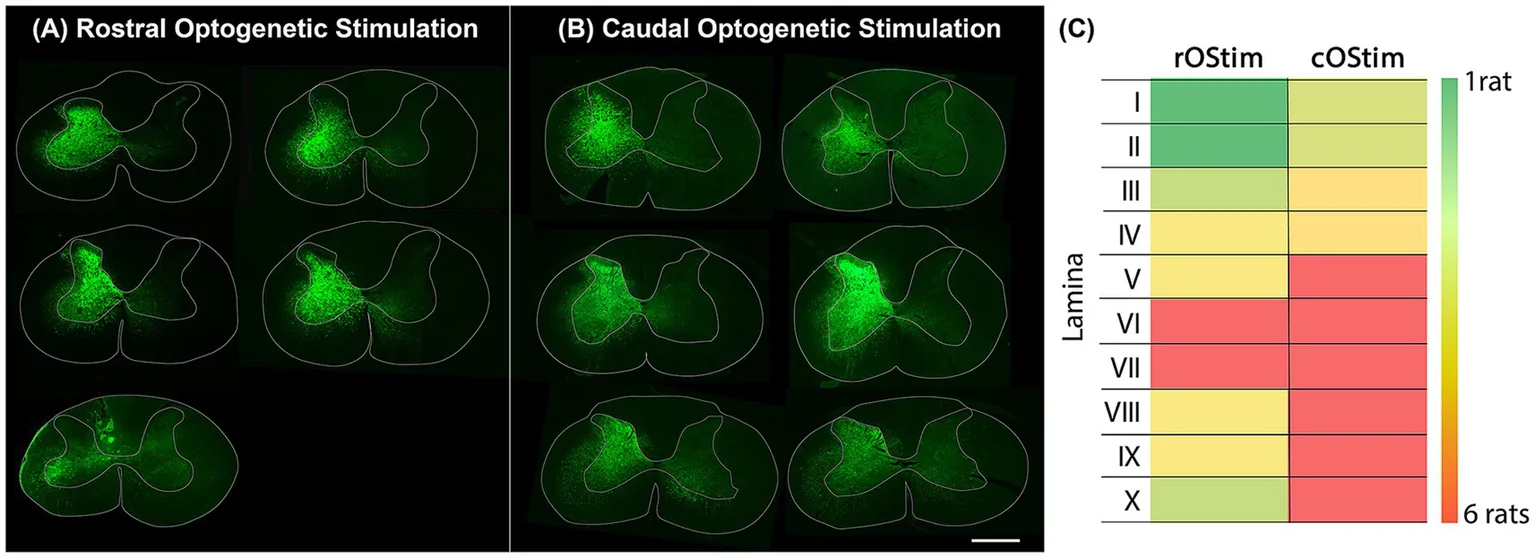
Optogenetic viral vector transduction. Representative images of the sections containing the greatest spread of viral transduction (green) for each stimulated rat are shown for the rostral **(A)** and caudal **(B)** optogenetic stimulation groups. Scale bar = 1,000 μm. **(C)** A heat map showing the proportion of rats containing viral transduction at each of the gray matter laminae is also presented.

### Lesion magnitudes were similar across groups

To ensure functional group differences were not due to differences in lesion magnitude, we compared the average percentage of spared tissue at the lesion epicenter for each group. There were no significant group differences (*p* = 0.501) (Figure 3A). The average percentage of tissue sparing was 81% ± 5%, 88% ± 3%, 82% ± 3%, and 86% ± 3% for the rostral sham, caudal sham, rostral stimulation, and caudal stimulation groups, respectively.

**Figure 3:**
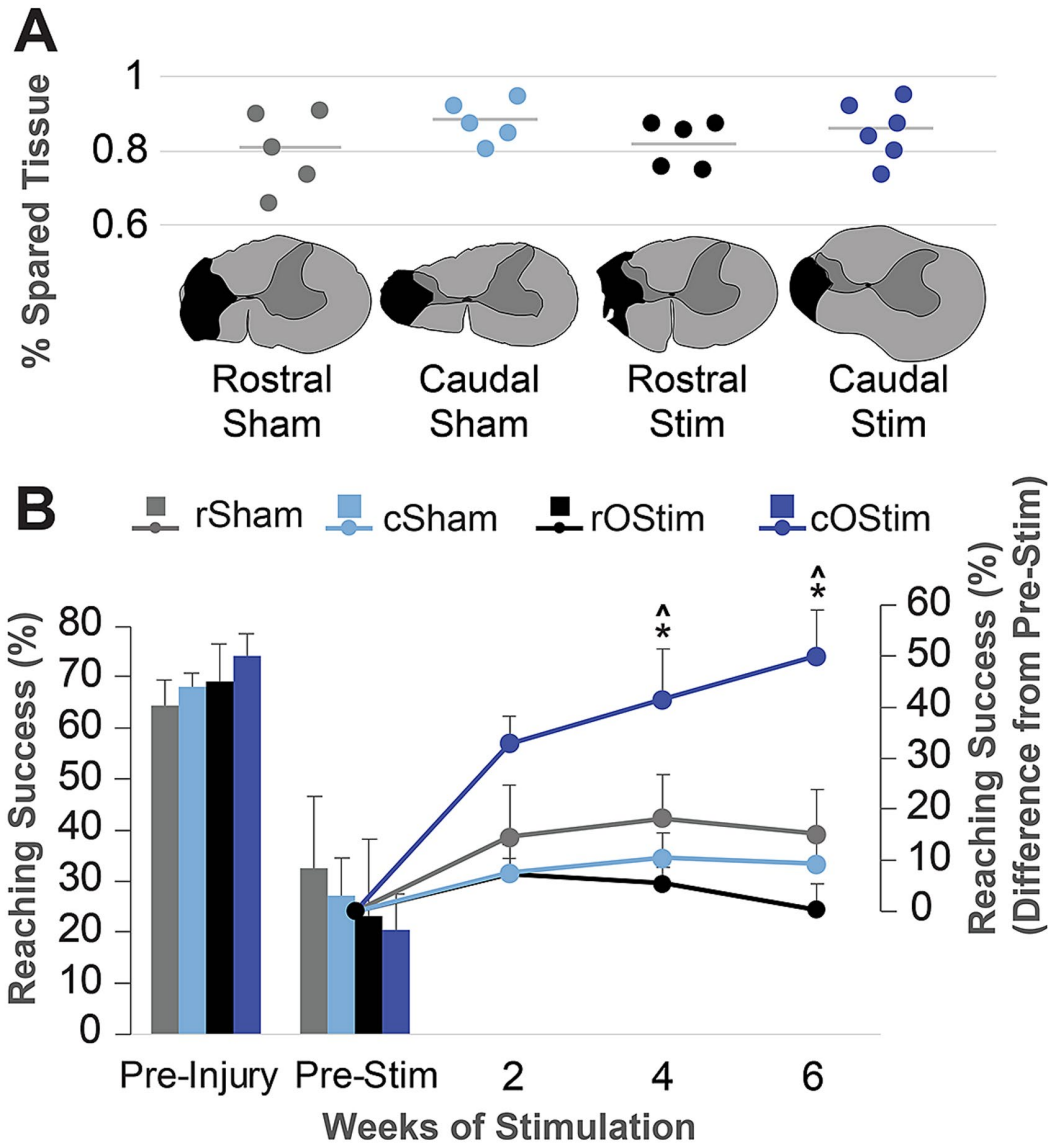
Lesion sparing and forelimb functional recovery. **(A)** Scatterplots depict the percentage of tissue sparing at the lesion epicenter for individual rats in each group. The horizontal lines show the group averages for each group. The black and white outlined drawings depict the median lesion epicenter for each group. **(B)** Reaching success for each group on the forelimb reaching task is also depicted. Bar graphs show the average reaching success (%) for each group prior to injury and during the week before stimulation began (5 weeks post-injury, “Pre-Stim”). The line graphs show the average reaching success after optogenetic stimulation began, normalized to the pre-stimulation week for each rat. *p < 0.05 when comparing caudal and rostral optogenetic stimulation groups, and ^^^*p* < 0.05 when comparing caudal sham and caudal optogenetic stimulation groups. rSham = rostral sham, cSham = caudal sham, rOStim = rostral optogenetic stimulation, cOStim = caudal optogenetic stimulation.

### Optogenetic stimulation caudal to the lesion promoted better recovery than stimulation rostral to the lesion

Prior to injury, all four groups had similar rates of reaching success (Figure 3B). Similarly, there were no group differences in reaching performance during the week prior to stimulation. This indicated that all groups had similar forelimb function prior to injury, as well as acutely after injury.

However, significant group differences were detected after 4 and 6 weeks of optogenetic stimulation (*p* = 0.031 and 0.007, respectively) (Figure 3B). Specifically, after 4 weeks of optogenetic stimulation, the caudally stimulated group had significantly higher reaching success (normalized to pre-stimulation performance; 38% ± 9%) than that of the rostrally stimulated group (5% ± 3%; *p* = 0.007). After 6 weeks of stimulation, reaching success increased to 45% ± 8% for the caudally stimulated group. This was significantly higher than the rostrally stimulated group, whose reaching success decreased to 0% ± 5% (*p* < 0.001). Caudal stimulation significantly outperformed rostral stimulation.

As previously reported (Mondello et al., 2023), the caudally stimulated group also had significantly higher reaching success compared with its sham group at 4 and 6 weeks of stimulation (*p* < 0.025). There were no significant differences in reaching success between the rostrally stimulated and sham groups at those same time periods (*p* = 0.372 and 0.707, respectively) (Figure 3B). Only caudally positioned stimulation had a therapeutic functional impact.

### Optogenetic stimulation caudal to the lesion enhances GAP43, while rostral stimulation does not

We compared the total area of GAP43 immunoreactivity, a marker for axonal growth, in all rats. Three regions were assessed: rostral to the injury, at the level of the injury, and caudal to the injury. Significant group differences were present at each of these levels (*p* < 0.015) (Figure 4). Rostral to the injury, the caudal stimulation group had significantly higher percent area of GAP43 compared to the rostrally stimulated group on both the ipsilateral and contralateral sides relative to the lesion (*p* = 0.001, *p* < 0.001, respectively) (Figure 4A).

**Figure 4:**
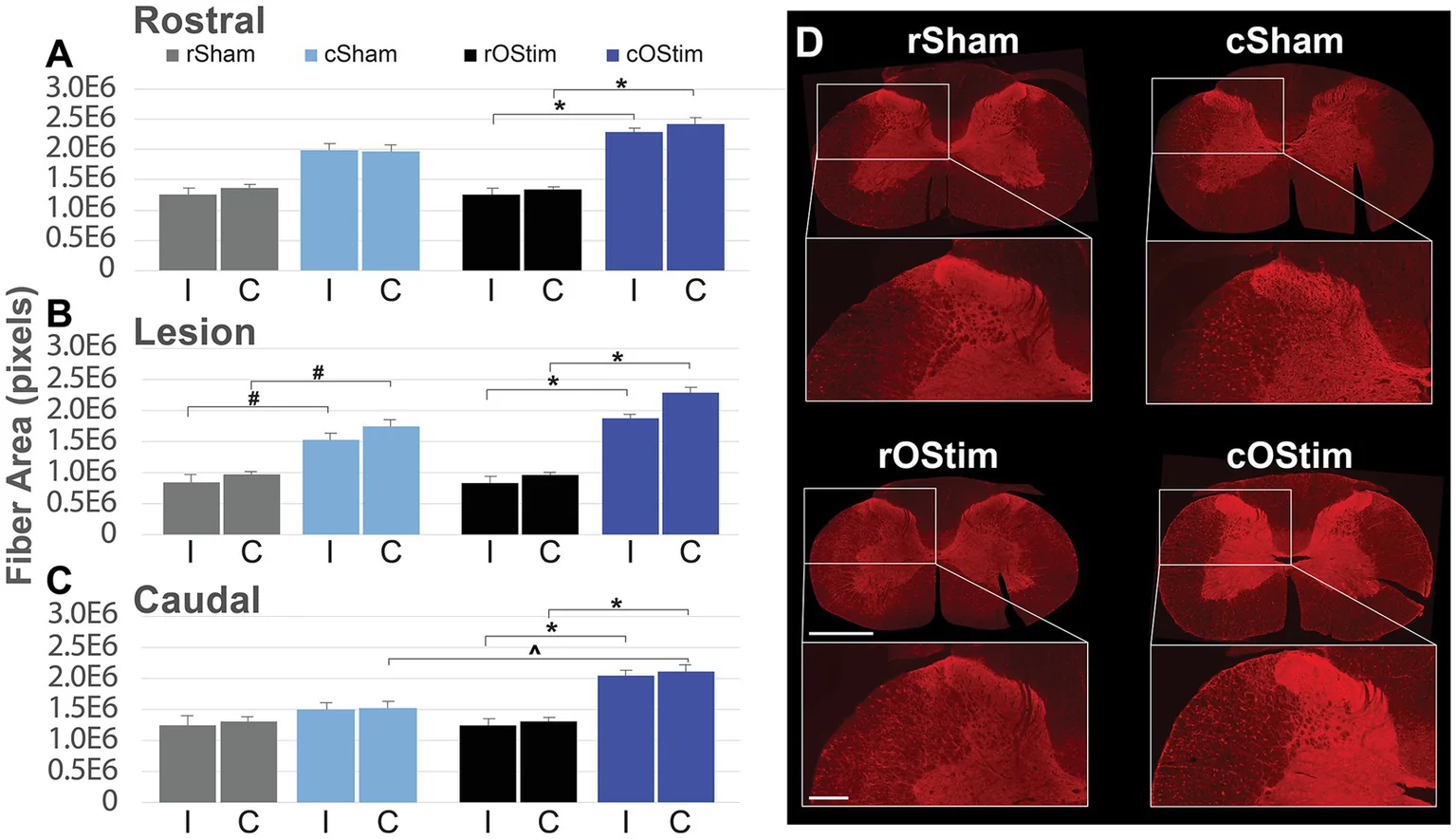
Growth-associated protein 43 (GAP43) plasticity. **(A)** Bar graphs show the percent area of GAP43-labeled axons rostral to the lesion, **(B)** at the level of the lesion, and **(C)** caudal to the lesion. These values are depicted for the rostral sham (rSham), caudal sham (cSham), rostral optogenetic stimulation (rOStim), and caudal optogenetic stimulation (cOStim) groups. I = ipsilateral to the lesion; C = contralateral to the lesion. *p < 0.05 when comparing caudal and rostral optogenetic stimulation groups, ^^^*p* < 0.05 when comparing caudal sham and caudal optogenetic stimulation groups, and ^#^*p* < 0.05 when comparing caudal and rostral sham groups. **(D)** Representative images of GAP43 labeling (red) within the caudal spinal cord are shown for each of the four groups. Scale bar for the low-magnification images = 1,000 μm. Insets show GAP43 expression in the ipsilateral dorsal gray matter at a higher magnification; scale bar = 300 μm.

A similar effect was seen at the level of the lesion, with the caudally stimulated group also having a significantly higher percent area of GAP43 compared to the rostrally stimulated group (ipsilateral, *p* = 0.006; contralateral, *p* = 0.004) (Figure 4B). Additionally, the caudal sham group had significantly higher GAP43 percent area than that in the rostral sham group (ipsilateral, *p* = 0.048; contralateral, *p* = 0.019).

In the region caudal to the lesion, the caudally stimulated group also had significantly higher percent area of GAP43 compared to the rostrally stimulated group (ipsilateral, *p* = 0.001; contralateral, *p* < 0.001) (Figure 4C). Additionally, in this location, caudally stimulated rats had significantly higher GAP43 percent area contralaterally compared to their shams (*p* = 0.033), similar to what we have previously reported (Mondello et al., 2023). In contrast, GAP43 percent area did not differ significantly between the rostrally stimulated and rostral sham groups in any region. Overall, these results indicate that caudal stimulation promoted more axonal growth than rostral stimulation. This aligns with the functional recovery results on the forelimb reaching task described above.

## Discussion

Here, we compare the effects of optogenetic spinal stimulation rostral and caudal to the lesion site on functional recovery and axonal sprouting after an SCI. Optogenetic stimulation caudal to the injury promoted significantly better functional recovery and more axonal sprouting than rostral stimulation. Rostral stimulation did not improve either outcome compared with its sham group, whereas caudal stimulation did enhance functional recovery and axonal growth compared with its sham counterpart.

The enhanced functional recovery observed in the caudal optogenetic stimulation group may be explained, at least in part, by the significantly greater GAP43 immunoreactivity, a marker of axonal growth, detected in the contralateral caudal spinal cord near the site of stimulation. Although previous work found more gray matter loss caudal to an SCI than rostral to it, the same study also found that caudal motoneurons retained high levels of acetylcholine and postsynaptic molecules (e.g., AMPAR1, NMDAR1). This indicates they are still capable of activation, synaptogenesis, and forming new circuitry (Yokota et al., 2019). Relatedly, a previous study presented evidence that optogenetic stimulation both attracts and guides axonal growth toward the location of stimulation. In zebrafish, axons closely followed the location of optogenetic stimulation across long distances, even as the location of stimulation was updated. This included guiding axons through and past inhibitory environments (Harris et al., 2020). Due to the location of enhanced GAP43 upregulation in the caudal optogenetic stimulation group (contralateral and caudal), these findings suggest that caudal optogenetic stimulation likely tapped into the preserved plastic capabilities of the caudal motoneuron populations. This likely both enhanced and attracted spared axonal pathways toward the site of stimulation, promoting new lesion-bypassing and recovery-promoting circuitry as described in our previous study (Mondello et al., 2023).

The lack of plasticity and recovery seen in the rostral stimulation group is unexpected, as previous studies suggest that physiological aspects of the rostral spinal cord may promote new plasticity after injury. For example, the segment immediately rostral to the spinal cord injury suffers less gray matter loss (Yokota et al., 2019) and has less upregulation of perineuronal net proteins that limit plasticity (Grycz et al., 2022) compared to segments caudal to the lesion. Furthermore, neuronal activation upregulates regenerative factors and growth factors of the activated neurons, which can promote axonal growth and plasticity (Mondello et al., 2014; Al-Majed et al., 2000; Al-Majed et al., 2000; Al-Majed et al., 2004). Therefore, stimulation of rostral neuronal populations could potentially lead to downstream axonal growth in a rostral-to-caudal fashion, with the benefit of bridging the lesion site. However, our results reveal that this does not occur with the presently studied paradigm.

The lack of improved therapeutic outcomes following rostral optogenetic stimulation could be due to challenges that are more pronounced in the rostral spinal cord, including ischemia–reperfusion, mitochondrial dysfunction, and oxidative stress, leading to more immediate degeneration (Ohnishi et al., 2021). These processes are thought to impair the rostral cord by limiting local oxygen and glucose delivery, reducing mitochondrial adenosine triphosphate (ATP) production, and generating reactive oxygen species that damage axonal and synaptic proteins, thereby blunting the regenerative response to stimulation. Neuroprotective strategies that target this pathophysiology, such as antioxidant or mitochondria-targeted therapies, may be needed to overcome these barriers and allow rostral stimulation to promote plasticity (Xing et al., 2025; Yu et al., 2023; Zhu et al., 2024). Additionally, a gene expression study found that after thoracic SCI, the rostral cord expressed fewer genes supporting neuronal fate and synaptic transmission than the caudal cord, suggesting that this region is less well suited for tissue regeneration (Cao et al., 2022). The delivery of growth factors such as brain-derived neurotrophic factor (BDNF) or neurotrophin-3 (NT-3) to the rostral cord after SCI could potentially improve this region’s regenerative capabilities (Zhou et al., 2003; Ramu et al., 2007; Weishaupt et al., 2013).

The more severe SCI-induced neuronal dysfunction rostral to the injury presents an additional issue (Cao et al., 2022): Due to the anatomical challenges of anchoring our implant at C2, the location of rostral stimulation was just one segment above the injury site in the present study. Any injury-related physiological challenges in the rostral cord could potentially extend into that location. It is possible that rostral stimulation two or more segments above the injury would have led to more plasticity and better functional outcomes. Indeed, other rostral therapeutic optogenetic stimulation studies that reported enhanced regeneration and functional recovery after an SCI had positioned optogenetic stimulation substantially farther up from the injury site in the motor cortex (Ma et al., 2025; Deng et al., 2021). While one of the studies optogenetically stimulated only glutamatergic neurons in rats (Deng et al., 2021), the other stimulated all neuronal cell types in mice and also reported improved plasticity and recovery (Ma et al., 2025). As such, it is difficult to interpret the potential impact of specific cell types on therapeutic outcomes, but this does highlight the therapeutic potential of long-distance upstream stimulation. It also emphasizes that optogenetic stimulation positioned farther rostrally in our injury model may promote better outcomes. Another study showed that upstream electrical stimulation of a denervated nerve led to quicker and more accurate muscle reinnervation, suggesting this result can occur in both the central and peripheral nervous systems (Al-Majed et al., 2000). One study attributed the improved therapeutic outcomes to increased activation of the JAK2/STAT3 pathway within the motor cortex (Ma et al., 2025), which has been shown to be involved in synaptic plasticity (Nicolas et al., 2012). This pathway should be evaluated within the spinal cord and motor cortex in the future.

Another important consideration is the location and directionality of the motor and sensory pathways relative to the site of stimulation. If optogenetic stimulation does have strong axon-attracting properties, as discussed above, *rostral* stimulation would attract motor pathway axons to grow *away* from their downstream neuronal targets located below the lesion rather than toward them. Therefore, a lack of appropriate directional guidance for motor circuits to grow toward downstream neuronal targets could contribute to the lack of recovery and axonal growth seen in the rostrally stimulated rats. This may also explain the success of caudal optogenetic stimulation, as it is correctly positioned at the downstream neuronal targets for motor pathways.

However, rostral stimulation presumably stimulates downstream *sensory* neuronal targets for disconnected sensory pathways. Due to the ascending directionality of these pathways, rostral stimulation could potentially promote the formation of a sensory lesion bridge. However, the lack of increased rostral axonal growth in the rostrally stimulated rats suggests this approach was somehow ineffective, potentially due to the rostral neuronal dysfunction described above (Ohnishi et al., 2021; Cao et al., 2022).

This study has several limitations. As mentioned above, the technical constraints for anchoring the μLED implant restricted the location of rostral stimulation to one spinal segment above the injury. Due to its proximity to the injury, this region could contain some injury-related tissue disruption that may negatively impact recovery. This differs from the caudal stimulation study, as the implant was positioned two segments below the injury site. Future research should explore whether stimulating potentially healthier tissue two or more segments rostral to the lesion or within the motor cortex itself would produce greater plasticity and functional benefits. Second, we did not directly measure neuroinflammatory markers, neural stem/progenitor cell proliferation, mitochondrial function, or oxidative stress within the rostral or caudal spinal cord relative to the lesion. As described above, spinal cord regions rostral to a lesion site may have greater dysfunction in these physiological processes after spinal cord injury (Ohnishi et al., 2021; Cao et al., 2022). Future studies should work to identify the type and degree of dysfunction within our SCI model to better delineate the underlying factors limiting the therapeutic efficacy of rostral optogenetic stimulation. Future work could then evaluate combinatorial treatments such as neuroprotective, pro-regenerative, or antioxidant co-interventions tailored to those findings. This, paired with a systematic comparison of different rostral stimulation sites, could help clarify why rostral stimulation was ineffective in the present study while also identifying strategies for improving optogenetic stimulation’s overall efficacy.

## Conclusion

Our findings indicate that optogenetic spinal stimulation one segment rostral to the lesion site, using our injury and stimulation model, does not enhance axonal growth or functional recovery. Optogenetic stimulation caudal to the lesion site is significantly more effective in improving axonal growth and functional recovery after SCI.

## Data Availability

The raw data supporting the conclusions of this article will be made available by the authors, without undue reservation.
